# Bis{bis­[2-(diisopropyl­phosphan­yl)phen­yl]phosphanido-κ^3^ *P*,*P*′,*P*′′}chloridonickel(II)

**DOI:** 10.1107/S1600536812044947

**Published:** 2012-11-07

**Authors:** Consiglia Tedesco, Marina Lamberti, Mina Mazzeo

**Affiliations:** a Dipartimento di Chimica e Biologia, Università di Salerno, via Ponte Don Melillo, I-84084 Fisciano, Italy

## Abstract

In the title compound, [Ni(C_24_H_36_P_3_)Cl], the Ni^II^ atom adopts a distorted square-planar geometry with the two neutral P atoms of the tridentate ligand *trans* to one another. Bond lengths and angles of the phosphide P atom feature a pyramidal geometry of the donor atom, which forms a single bond with the Ni^II^ atom, retaining a stereochemically active lone pair.

## Related literature

For related structures, see: Boro *et al.* (2008 ▶); Liang *et al.* (2006 ▶); Mazzeo *et al.* (2008 ▶, 2011 ▶).
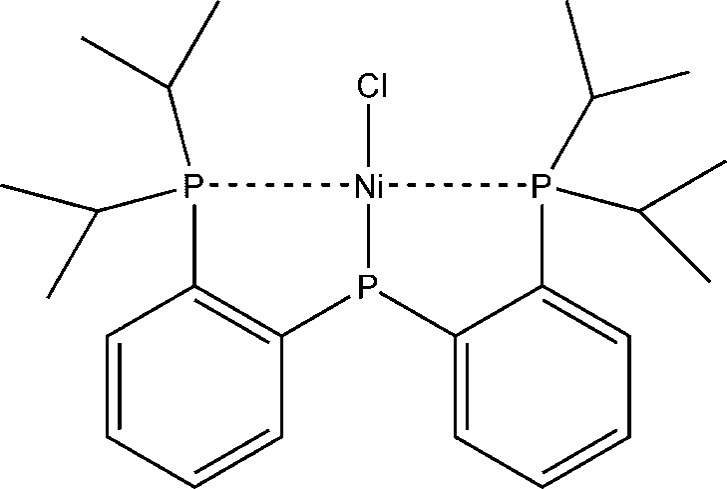


## Experimental

### 

#### Crystal data

[Ni(C_24_H_36_P_3_)Cl]
*M* *_r_* = 511.60
Monoclinic, 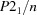

*a* = 13.752 (3) Å
*b* = 11.978 (2) Å
*c* = 15.554 (4) Å
β = 101.043 (17)°
*V* = 2514.7 (9) Å^3^
*Z* = 4
Mo *K*α radiation
μ = 1.08 mm^−1^
*T* = 100 K
0.30 × 0.25 × 0.15 mm

#### Data collection

Rigaku Mercury2 diffractometer
Absorption correction: multi-scan (Blessing, 1995 ▶) *T*_min_ = 0.725, *T*_max_ = 0.875
36493 measured reflections
10236 independent reflections
7497 reflections with *I* > 2σ(*I*)
*R*_int_ = 0.077

#### Refinement

*R*[*F* ^2^ > 2σ(*F* ^2^)] = 0.041
*wR*(*F* ^2^) = 0.108
*S* = 1.00
10236 reflections
270 parameters
H-atom parameters constrained
Δρ_max_ = 1.04 e Å^−3^
Δρ_min_ = −0.46 e Å^−3^

### 

Data collection: *CrystalClear* (Rigaku, 2007 ▶); cell refinement: *CrystalClear*; data reduction: *CrystalClear*; program(s) used to solve structure: *SHELXS97* (Sheldrick, 2008 ▶); program(s) used to refine structure: *SHELXL97* (Sheldrick, 2008 ▶); molecular graphics: *ORTEP-3* (Farrugia, 1997 ▶); software used to prepare material for publication: *PLATON* (Spek, 2009 ▶).

## Supplementary Material

Crystal structure: contains datablock(s) global, I. DOI: 10.1107/S1600536812044947/im2406sup1.cif

Structure factors: contains datablock(s) I. DOI: 10.1107/S1600536812044947/im2406Isup2.hkl

Additional supplementary materials:  crystallographic information; 3D view; checkCIF report

## Figures and Tables

**Fig. 1.**
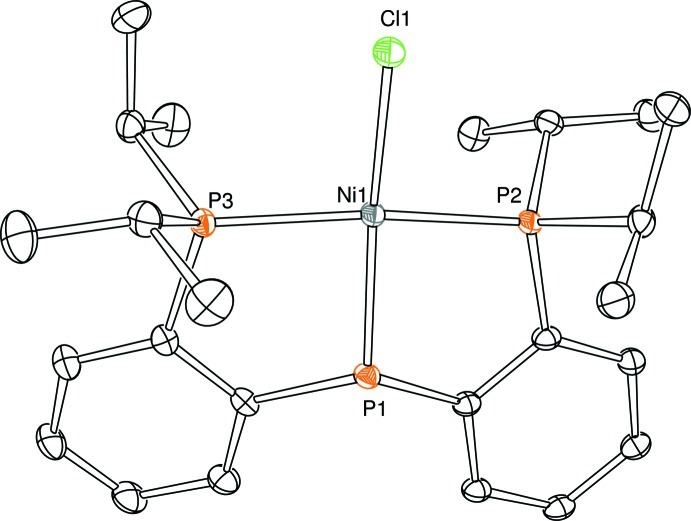
A view of (1). Displacement ellipsoids are drawn at the 50% probability level. Hydrogen atoms are omitted for clarity.

## supplementary crystallographic information

### Comment

The solid-state structure of the title compound [P(o—C_6_H_4_P(CH(CH_3_)_2_)_2_]NiCl (see Scheme 1) confirms the tridentate feature of the phosphido diphosphine ligand. The coordination environment around Ni (II) is approximately square planar with the chloride ligand being *trans* to the phosphido phosphorous atom. The deviation from the idealized square planar geometry is primarily caused by the chelate PPP constraint with a P(3)—Ni(1)—P(2) angle of 164.81 (2)°. The Ni—Cl distance (2.2230 (6) Å) is appreciably longer than that observed in the related [iPr-PNP]Ni—Cl complex (2.1834 (6) Å), Liang *et al.*, 2006) but shorter than that observed in the complex [tertBut-PCP]NiCl (2.2317 (5) Å, Boro *et al.*, 2008), suggesting that the *trans* influence of the phosphido donor is larger than that of the corresponding amido ligand but less than that of the anionic aryl carbon. Bond distances and angles (between 108.17 (5)° and 111.03 (7)°) of the phosphido phosphorous atom suggest a pyramidal geometry of the donor atom in which the phosphorous donor forms a single bond with the nickel centre and retains a stereochemically active lone pair. This structure is reminiscent of those reported for the related platinum and palladium complexes (Mazzeo *et al.*, 2008; Mazzeo *et al.*, 2011).

### Experimental

To a suspension of NiCl_2_ (0.216 g; 1.67 mmol) in THF (5 ml) a solution of the ligand (0.700 g; 1.67 mmol) in THF (15 ml) and a solution of HNEtiPr_2_ (0.309 g, 2.39 mmol) in 2 ml of THF were quickly added, at room temperature. The color of the reaction mixture quickly turned to red purple and the resulting slurry was stirred at 50 °C for 2 h. The resulting deep purple solution was cooled to room temperature and volatile material was removed *in vacuo* affording a red purple residue. This crude product was extracted with benzene (15 ml) and filtered through celite on a sintered-glass frit. The solvent was again removed under reduced pressure. The obtained red solid was washed with methanol (2 × 3 ml), with petroleum ether (2 × 5 ml) and then dried *in vacuo* to give the desidered product as analytically pure compound (0.600 g, yield 70%). Crystals for X-ray analysis were obtained *via* vapor diffusion of petroleum ether into a THF solution of the complex.

### Refinement

All H atoms were placed geometrically and treated as riding on their parent atoms with O—H = 0.82 Å [U_iso_(H) = 1.5 U_eq_(O)], C—H = 0.97 (methyl) Å [U_iso_(H) = 1.5 U_eq_(C)], and C—H = 0.93 (aromatic and methine) Å [U_iso_(H) = 1.2 U_eq_(C)].

### Crystal data

**Table:**

[Ni(C_24_H_36_P_3_)Cl]	*F*(000) = 1080
*M**_r_* = 511.60	*D*_x_ = 1.351 Mg m^−^^3^
Monoclinic, *P*2_1_/*n*	Mo *K*α radiation, λ = 0.71073 Å
Hall symbol: -P 2yn	Cell parameters from 10236 reflections
*a* = 13.752 (3) Å	θ = 1.8–38.3°
*b* = 11.978 (2) Å	µ = 1.08 mm^−^^1^
*c* = 15.554 (4) Å	*T* = 100 K
β = 101.043 (17)°	Platelet, red
*V* = 2514.7 (9) Å^3^	0.30 × 0.25 × 0.15 mm
*Z* = 4	

### Data collection

**Table:**

Rigaku Mercury2 diffractometer	10236 independent reflections
Radiation source: fine-focus sealed tube	7497 reflections with *I* > 2σ(*I*)
Graphite monochromator	*R*_int_ = 0.077
Detector resolution: 13.6612 pixels mm^-1^	θ_max_ = 38.3°, θ_min_ = 1.8°
ω scans	*h* = −21→18
Absorption correction: multi-scan (Blessing, 1995)	*k* = −20→20
*T*_min_ = 0.725, *T*_max_ = 0.875	*l* = −26→26
36493 measured reflections	

### Refinement

**Table:**

Refinement on *F*^2^	Primary atom site location: structure-invariant direct methods
Least-squares matrix: full	Secondary atom site location: difference Fourier map
*R*[*F*^2^ > 2σ(*F*^2^)] = 0.041	Hydrogen site location: inferred from neighbouring sites
*wR*(*F*^2^) = 0.108	H-atom parameters constrained
*S* = 1.00	*w* = 1/[σ^2^(*F*_o_^2^) + (0.0583*P*)^2^] where *P* = (*F*_o_^2^ + 2*F*_c_^2^)/3
10236 reflections	(Δ/σ)_max_ = 0.001
270 parameters	Δρ_max_ = 1.04 e Å^−^^3^
0 restraints	Δρ_min_ = −0.46 e Å^−^^3^

### Special details

**Table:**

Experimental. ^1^H NMR (300 MHz; benzene-d6): δ 7.83(dd, 2 H, ^2^JP—H = 8 Hz, ^1^JH—H = 2 Hz, Ar—H), 7.13 (t, 2H, ^2^JP—H = 8 Hz, Ar—H), 7.00 (b, 2H, Ar—H), 6.93(t, 2H, ^2^JP—H= 7 Hz Ar—H,), 2.52 (m, 4H, CH(CH_3_)_2_), 1.49 (dd, 12 H, CH(CH_3_)_2_, J = 16 and 7 Hz), 1.05 (dd, 12H, J = 16 and 7 Hz CH(CH_3_)_2_).^13^C{^1^H} NMR (75.409 MHz; benzene-d6): δ 148.7, 146.9, 131.7, 131.3, 129.1, 125.9, 26.07(t, J=11 Hz, CH(CH_3_)_2_), 19.70 (bs, CH(CH_3_)_2_),18.62 (s, CH(CH_3_)_2_).^31^P{^1^H} NMR (121.4 MHz; benzene-d6): δ 115.32 (t, 1P, ^3^JP—P = 9 Hz), 54.02 (d, 2P, ^3^JP—P = 9 Hz).
Geometry. All e.s.d.'s (except the e.s.d. in the dihedral angle between two l.s. planes) are estimated using the full covariance matrix. The cell e.s.d.'s are taken into account individually in the estimation of e.s.d.'s in distances, angles and torsion angles; correlations between e.s.d.'s in cell parameters are only used when they are defined by crystal symmetry. An approximate (isotropic) treatment of cell e.s.d.'s is used for estimating e.s.d.'s involving l.s. planes.
Refinement. Refinement of *F*^2^ against ALL reflections. The weighted *R*-factor *wR* and goodness of fit *S* are based on *F*^2^, conventional *R*-factors *R* are based on *F*, with *F* set to zero for negative *F*^2^. The threshold expression of *F*^2^ > σ(*F*^2^) is used only for calculating *R*-factors(gt) *etc*. and is not relevant to the choice of reflections for refinement. *R*-factors based on *F*^2^ are statistically about twice as large as those based on *F*, and *R*- factors based on ALL data will be even larger.

### Fractional atomic coordinates and isotropic or equivalent isotropic displacement parameters (Å^2^)

**Table:**

	*x*	*y*	*z*	*U*_iso_*/*U*_eq_	
Ni1	0.507688 (15)	0.862502 (15)	0.234394 (12)	0.01174 (6)	
P1	0.38852 (3)	0.76794 (3)	0.15893 (2)	0.01232 (8)	
P2	0.60305 (3)	0.77839 (3)	0.15774 (2)	0.01240 (8)	
P3	0.39159 (3)	0.96155 (3)	0.27720 (2)	0.01268 (8)	
Cl1	0.63222 (3)	0.92364 (4)	0.33635 (3)	0.02309 (9)	
C1	0.27837 (12)	0.85825 (12)	0.13536 (10)	0.0144 (3)	
C2	0.19587 (13)	0.84451 (13)	0.06790 (11)	0.0193 (3)	
H2	0.1978	0.7914	0.0229	0.023*	
C3	0.11074 (13)	0.90847 (14)	0.06644 (11)	0.0224 (3)	
H3	0.0555	0.8995	0.0199	0.027*	
C4	0.10613 (13)	0.98523 (14)	0.13266 (12)	0.0229 (3)	
H4	0.0473	1.0271	0.1321	0.027*	
C5	0.18779 (13)	1.00058 (14)	0.19964 (11)	0.0200 (3)	
H5	0.1848	1.0532	0.2448	0.024*	
C6	0.27443 (12)	0.93868 (12)	0.20071 (10)	0.0153 (3)	
C7	0.37738 (13)	0.92346 (13)	0.38917 (10)	0.0184 (3)	
H7	0.4392	0.9485	0.4294	0.022*	
C8	0.37380 (16)	0.79562 (15)	0.39703 (12)	0.0274 (4)	
H8A	0.3729	0.7751	0.4579	0.041*	
H8B	0.4324	0.7631	0.3794	0.041*	
H8C	0.3139	0.7672	0.3588	0.041*	
C9	0.29155 (16)	0.97945 (17)	0.42077 (12)	0.0291 (4)	
H9A	0.2288	0.9522	0.3863	0.044*	
H9B	0.2958	1.0605	0.4136	0.044*	
H9C	0.2946	0.9618	0.4828	0.044*	
C10	0.40201 (13)	1.11551 (12)	0.27837 (11)	0.0184 (3)	
H10	0.3379	1.1464	0.2893	0.022*	
C11	0.48453 (15)	1.15913 (14)	0.35091 (13)	0.0277 (4)	
H11A	0.5482	1.1277	0.3433	0.042*	
H11B	0.4710	1.1368	0.4081	0.042*	
H11C	0.4872	1.2407	0.3477	0.042*	
C12	0.41603 (17)	1.15781 (15)	0.18882 (13)	0.0295 (4)	
H12A	0.4133	1.2396	0.1879	0.044*	
H12B	0.3633	1.1279	0.1432	0.044*	
H12C	0.4805	1.1331	0.1779	0.044*	
C13	0.42472 (12)	0.72062 (11)	0.05737 (9)	0.0129 (3)	
C14	0.36210 (12)	0.67045 (12)	−0.01409 (9)	0.0151 (3)	
H14	0.2923	0.6732	−0.0182	0.018*	
C15	0.40146 (13)	0.61688 (12)	−0.07888 (10)	0.0163 (3)	
H15	0.3581	0.5855	−0.1278	0.020*	
C16	0.50366 (13)	0.60846 (12)	−0.07316 (10)	0.0167 (3)	
H16	0.5299	0.5706	−0.1173	0.020*	
C17	0.56680 (13)	0.65619 (12)	−0.00203 (10)	0.0161 (3)	
H17	0.6365	0.6496	0.0031	0.019*	
C18	0.52782 (12)	0.71385 (11)	0.06193 (9)	0.0139 (3)	
C19	0.68016 (13)	0.66106 (13)	0.20976 (10)	0.0171 (3)	
H19	0.7007	0.6155	0.1624	0.021*	
C20	0.61433 (14)	0.58829 (13)	0.25608 (11)	0.0220 (3)	
H20A	0.6494	0.5191	0.2763	0.033*	
H20B	0.5528	0.5704	0.2152	0.033*	
H20C	0.5987	0.6289	0.3064	0.033*	
C21	0.77391 (13)	0.69711 (14)	0.27367 (11)	0.0228 (4)	
H21A	0.7554	0.7391	0.3222	0.034*	
H21B	0.8144	0.7445	0.2430	0.034*	
H21C	0.8120	0.6309	0.2967	0.034*	
C22	0.68475 (13)	0.88108 (13)	0.11899 (10)	0.0168 (3)	
H22	0.7229	0.9188	0.1723	0.020*	
C23	0.76138 (14)	0.83460 (15)	0.06823 (12)	0.0228 (3)	
H23A	0.7276	0.8092	0.0103	0.034*	
H23B	0.7964	0.7717	0.1005	0.034*	
H23C	0.8090	0.8933	0.0615	0.034*	
C24	0.62145 (14)	0.97121 (14)	0.06549 (11)	0.0225 (3)	
H24A	0.6647	1.0287	0.0483	0.034*	
H24B	0.5776	1.0051	0.1010	0.034*	
H24C	0.5815	0.9376	0.0129	0.034*	

### Atomic displacement parameters (Å^2^)

**Table:**

	*U*^11^	*U*^22^	*U*^33^	*U*^12^	*U*^13^	*U*^23^
Ni1	0.00990 (11)	0.01376 (8)	0.01101 (8)	0.00084 (6)	0.00062 (7)	−0.00213 (6)
P1	0.01050 (19)	0.01411 (15)	0.01187 (15)	−0.00029 (13)	0.00091 (14)	−0.00044 (11)
P2	0.00955 (19)	0.01467 (15)	0.01221 (15)	0.00058 (13)	0.00013 (14)	−0.00314 (12)
P3	0.0121 (2)	0.01420 (15)	0.01216 (15)	0.00082 (13)	0.00324 (14)	−0.00029 (12)
Cl1	0.0147 (2)	0.02913 (18)	0.02231 (17)	0.00337 (15)	−0.00438 (15)	−0.01218 (14)
C1	0.0106 (7)	0.0174 (6)	0.0154 (6)	−0.0002 (5)	0.0026 (5)	0.0020 (5)
C2	0.0145 (8)	0.0220 (7)	0.0198 (7)	0.0004 (6)	−0.0012 (6)	−0.0002 (5)
C3	0.0141 (8)	0.0259 (7)	0.0248 (8)	0.0013 (6)	−0.0028 (6)	0.0016 (6)
C4	0.0130 (8)	0.0260 (7)	0.0291 (8)	0.0059 (6)	0.0024 (7)	0.0010 (6)
C5	0.0158 (9)	0.0224 (7)	0.0226 (7)	0.0035 (6)	0.0057 (6)	−0.0003 (6)
C6	0.0138 (8)	0.0169 (6)	0.0160 (6)	0.0011 (5)	0.0044 (6)	0.0010 (5)
C7	0.0195 (9)	0.0225 (7)	0.0143 (6)	−0.0018 (6)	0.0060 (6)	0.0003 (5)
C8	0.0383 (12)	0.0243 (7)	0.0223 (8)	−0.0035 (7)	0.0125 (8)	0.0064 (6)
C9	0.0308 (11)	0.0387 (10)	0.0215 (8)	0.0054 (8)	0.0141 (8)	0.0018 (7)
C10	0.0182 (9)	0.0149 (6)	0.0233 (7)	0.0013 (5)	0.0068 (6)	0.0001 (5)
C11	0.0269 (10)	0.0178 (7)	0.0366 (10)	−0.0021 (7)	0.0017 (8)	−0.0084 (6)
C12	0.0382 (12)	0.0216 (7)	0.0316 (9)	0.0013 (7)	0.0138 (9)	0.0082 (7)
C13	0.0136 (7)	0.0131 (5)	0.0109 (5)	−0.0009 (5)	−0.0004 (5)	−0.0001 (4)
C14	0.0134 (8)	0.0152 (5)	0.0151 (6)	−0.0014 (5)	−0.0011 (6)	−0.0004 (5)
C15	0.0189 (8)	0.0151 (6)	0.0128 (6)	−0.0023 (5)	−0.0020 (6)	−0.0011 (4)
C16	0.0200 (9)	0.0165 (6)	0.0131 (6)	−0.0013 (6)	0.0022 (6)	−0.0030 (5)
C17	0.0141 (8)	0.0179 (6)	0.0157 (6)	−0.0003 (5)	0.0016 (6)	−0.0035 (5)
C18	0.0138 (8)	0.0143 (5)	0.0124 (6)	−0.0013 (5)	−0.0008 (5)	−0.0014 (4)
C19	0.0153 (8)	0.0184 (6)	0.0159 (6)	0.0050 (5)	−0.0014 (6)	−0.0035 (5)
C20	0.0216 (9)	0.0192 (6)	0.0232 (7)	0.0016 (6)	−0.0007 (7)	0.0009 (6)
C21	0.0170 (9)	0.0253 (7)	0.0225 (7)	0.0033 (6)	−0.0051 (6)	−0.0029 (6)
C22	0.0128 (8)	0.0200 (6)	0.0178 (6)	−0.0027 (5)	0.0035 (6)	−0.0043 (5)
C23	0.0166 (9)	0.0291 (8)	0.0241 (8)	−0.0018 (7)	0.0077 (7)	−0.0038 (6)
C24	0.0249 (10)	0.0209 (7)	0.0226 (7)	0.0009 (6)	0.0067 (7)	0.0011 (6)

### Geometric parameters (Å, º)

**Table:**

Ni1—P1	2.1469 (6)	C11—H11A	0.9800
Ni1—P2	2.1807 (6)	C11—H11B	0.9800
Ni1—P3	2.1925 (6)	C11—H11C	0.9800
Ni1—Cl1	2.2234 (6)	C12—H12A	0.9800
P1—C13	1.8347 (16)	C12—H12B	0.9800
P1—C1	1.8402 (16)	C12—H12C	0.9800
P2—C18	1.8160 (14)	C13—C14	1.4041 (19)
P2—C22	1.8434 (17)	C13—C18	1.408 (2)
P2—C19	1.8508 (15)	C14—C15	1.389 (2)
P3—C6	1.8307 (16)	C14—H14	0.9500
P3—C7	1.8467 (17)	C15—C16	1.395 (2)
P3—C10	1.8497 (16)	C15—H15	0.9500
C1—C2	1.400 (2)	C16—C17	1.392 (2)
C1—C6	1.409 (2)	C16—H16	0.9500
C2—C3	1.396 (2)	C17—C18	1.399 (2)
C2—H2	0.9500	C17—H17	0.9500
C3—C4	1.391 (3)	C19—C20	1.533 (3)
C3—H3	0.9500	C19—C21	1.532 (2)
C4—C5	1.390 (2)	C19—H19	1.0000
C4—H4	0.9500	C20—H20A	0.9800
C5—C6	1.401 (2)	C20—H20B	0.9800
C5—H5	0.9500	C20—H20C	0.9800
C7—C9	1.519 (3)	C21—H21A	0.9800
C7—C8	1.538 (2)	C21—H21B	0.9800
C7—H7	1.0000	C21—H21C	0.9800
C8—H8A	0.9800	C22—C24	1.529 (2)
C8—H8B	0.9800	C22—C23	1.537 (2)
C8—H8C	0.9800	C22—H22	1.0000
C9—H9A	0.9800	C23—H23A	0.9800
C9—H9B	0.9800	C23—H23B	0.9800
C9—H9C	0.9800	C23—H23C	0.9800
C10—C12	1.529 (3)	C24—H24A	0.9800
C10—C11	1.531 (2)	C24—H24B	0.9800
C10—H10	1.0000	C24—H24C	0.9800
			
P1—Ni1—P2	86.18 (2)	H11A—C11—H11B	109.5
P1—Ni1—P3	85.84 (2)	C10—C11—H11C	109.5
P2—Ni1—P3	164.837 (17)	H11A—C11—H11C	109.5
P1—Ni1—Cl1	165.171 (19)	H11B—C11—H11C	109.5
P2—Ni1—Cl1	94.61 (2)	C10—C12—H12A	109.5
P3—Ni1—Cl1	96.40 (2)	C10—C12—H12B	109.5
C13—P1—C1	111.03 (7)	H12A—C12—H12B	109.5
C13—P1—Ni1	109.22 (5)	C10—C12—H12C	109.5
C1—P1—Ni1	108.17 (5)	H12A—C12—H12C	109.5
C18—P2—C22	107.63 (7)	H12B—C12—H12C	109.5
C18—P2—C19	102.95 (7)	C14—C13—C18	118.31 (14)
C22—P2—C19	108.39 (8)	C14—C13—P1	125.98 (13)
C18—P2—Ni1	109.73 (6)	C18—C13—P1	114.26 (10)
C22—P2—Ni1	109.78 (5)	C15—C14—C13	120.46 (16)
C19—P2—Ni1	117.78 (6)	C15—C14—H14	119.8
C6—P3—C7	109.69 (8)	C13—C14—H14	119.8
C6—P3—C10	102.27 (7)	C14—C15—C16	121.00 (13)
C7—P3—C10	105.01 (8)	C14—C15—H15	119.5
C6—P3—Ni1	108.85 (6)	C16—C15—H15	119.5
C7—P3—Ni1	111.56 (6)	C17—C16—C15	119.25 (15)
C10—P3—Ni1	118.88 (6)	C17—C16—H16	120.4
C2—C1—C6	118.91 (15)	C15—C16—H16	120.4
C2—C1—P1	126.75 (12)	C16—C17—C18	120.16 (16)
C6—C1—P1	113.71 (11)	C16—C17—H17	119.9
C3—C2—C1	120.31 (15)	C18—C17—H17	119.9
C3—C2—H2	119.8	C17—C18—C13	120.76 (13)
C1—C2—H2	119.8	C17—C18—P2	123.91 (13)
C4—C3—C2	120.51 (15)	C13—C18—P2	115.26 (11)
C4—C3—H3	119.7	C20—C19—C21	110.78 (13)
C2—C3—H3	119.7	C20—C19—P2	107.27 (12)
C3—C4—C5	119.83 (16)	C21—C19—P2	114.22 (11)
C3—C4—H4	120.1	C20—C19—H19	108.1
C5—C4—H4	120.1	C21—C19—H19	108.1
C4—C5—C6	120.15 (16)	P2—C19—H19	108.1
C4—C5—H5	119.9	C19—C20—H20A	109.5
C6—C5—H5	119.9	C19—C20—H20B	109.5
C5—C6—C1	120.23 (14)	H20A—C20—H20B	109.5
C5—C6—P3	124.70 (12)	C19—C20—H20C	109.5
C1—C6—P3	114.86 (12)	H20A—C20—H20C	109.5
C9—C7—C8	112.05 (16)	H20B—C20—H20C	109.5
C9—C7—P3	115.17 (12)	C19—C21—H21A	109.5
C8—C7—P3	109.37 (11)	C19—C21—H21B	109.5
C9—C7—H7	106.6	H21A—C21—H21B	109.5
C8—C7—H7	106.6	C19—C21—H21C	109.5
P3—C7—H7	106.6	H21A—C21—H21C	109.5
C7—C8—H8A	109.5	H21B—C21—H21C	109.5
C7—C8—H8B	109.5	C24—C22—C23	110.55 (14)
H8A—C8—H8B	109.5	C24—C22—P2	109.27 (12)
C7—C8—H8C	109.5	C23—C22—P2	116.55 (11)
H8A—C8—H8C	109.5	C24—C22—H22	106.6
H8B—C8—H8C	109.5	C23—C22—H22	106.6
C7—C9—H9A	109.5	P2—C22—H22	106.6
C7—C9—H9B	109.5	C22—C23—H23A	109.5
H9A—C9—H9B	109.5	C22—C23—H23B	109.5
C7—C9—H9C	109.5	H23A—C23—H23B	109.5
H9A—C9—H9C	109.5	C22—C23—H23C	109.5
H9B—C9—H9C	109.5	H23A—C23—H23C	109.5
C12—C10—C11	110.70 (16)	H23B—C23—H23C	109.5
C12—C10—P3	110.15 (12)	C22—C24—H24A	109.5
C11—C10—P3	113.12 (11)	C22—C24—H24B	109.5
C12—C10—H10	107.5	H24A—C24—H24B	109.5
C11—C10—H10	107.5	C22—C24—H24C	109.5
P3—C10—H10	107.5	H24A—C24—H24C	109.5
C10—C11—H11A	109.5	H24B—C24—H24C	109.5
C10—C11—H11B	109.5		
			
P2—Ni1—P1—C13	−20.53 (5)	C6—P3—C7—C9	−53.35 (15)
P3—Ni1—P1—C13	146.57 (5)	C10—P3—C7—C9	55.90 (15)
Cl1—Ni1—P1—C13	−114.11 (8)	Ni1—P3—C7—C9	−174.04 (11)
P2—Ni1—P1—C1	−141.48 (6)	C6—P3—C7—C8	73.88 (14)
P3—Ni1—P1—C1	25.62 (6)	C10—P3—C7—C8	−176.87 (13)
Cl1—Ni1—P1—C1	124.94 (9)	Ni1—P3—C7—C8	−46.82 (14)
P1—Ni1—P2—C18	15.30 (5)	C6—P3—C10—C12	−66.43 (15)
P3—Ni1—P2—C18	−43.06 (9)	C7—P3—C10—C12	179.03 (13)
Cl1—Ni1—P2—C18	−179.54 (5)	Ni1—P3—C10—C12	53.41 (15)
P1—Ni1—P2—C22	133.40 (6)	C6—P3—C10—C11	169.10 (14)
P3—Ni1—P2—C22	75.03 (9)	C7—P3—C10—C11	54.56 (16)
Cl1—Ni1—P2—C22	−61.45 (6)	Ni1—P3—C10—C11	−71.06 (15)
P1—Ni1—P2—C19	−101.96 (6)	C1—P1—C13—C14	−51.18 (14)
P3—Ni1—P2—C19	−160.33 (8)	Ni1—P1—C13—C14	−170.37 (11)
Cl1—Ni1—P2—C19	63.19 (6)	C1—P1—C13—C18	142.91 (11)
P1—Ni1—P3—C6	−18.46 (5)	Ni1—P1—C13—C18	23.72 (11)
P2—Ni1—P3—C6	39.95 (9)	C18—C13—C14—C15	−0.8 (2)
Cl1—Ni1—P3—C6	176.27 (5)	P1—C13—C14—C15	−166.18 (11)
P1—Ni1—P3—C7	102.73 (6)	C13—C14—C15—C16	2.1 (2)
P2—Ni1—P3—C7	161.13 (8)	C14—C15—C16—C17	−1.0 (2)
Cl1—Ni1—P3—C7	−62.55 (6)	C15—C16—C17—C18	−1.3 (2)
P1—Ni1—P3—C10	−134.87 (6)	C16—C17—C18—C13	2.6 (2)
P2—Ni1—P3—C10	−76.46 (9)	C16—C17—C18—P2	179.52 (12)
Cl1—Ni1—P3—C10	59.86 (6)	C14—C13—C18—C17	−1.6 (2)
C13—P1—C1—C2	38.93 (17)	P1—C13—C18—C17	165.51 (11)
Ni1—P1—C1—C2	158.75 (14)	C14—C13—C18—P2	−178.71 (10)
C13—P1—C1—C6	−150.31 (12)	P1—C13—C18—P2	−11.64 (14)
Ni1—P1—C1—C6	−30.49 (13)	C22—P2—C18—C17	58.57 (14)
C6—C1—C2—C3	−1.0 (2)	C19—P2—C18—C17	−55.81 (15)
P1—C1—C2—C3	169.38 (14)	Ni1—P2—C18—C17	177.99 (11)
C1—C2—C3—C4	−1.1 (3)	C22—P2—C18—C13	−124.38 (11)
C2—C3—C4—C5	1.7 (3)	C19—P2—C18—C13	121.24 (12)
C3—C4—C5—C6	−0.2 (3)	Ni1—P2—C18—C13	−4.95 (12)
C4—C5—C6—C1	−1.8 (2)	C18—P2—C19—C20	−76.47 (12)
C4—C5—C6—P3	172.58 (14)	C22—P2—C19—C20	169.71 (10)
C2—C1—C6—C5	2.4 (2)	Ni1—P2—C19—C20	44.38 (11)
P1—C1—C6—C5	−169.15 (13)	C18—P2—C19—C21	160.36 (13)
C2—C1—C6—P3	−172.53 (12)	C22—P2—C19—C21	46.54 (15)
P1—C1—C6—P3	15.91 (16)	Ni1—P2—C19—C21	−78.80 (14)
C7—P3—C6—C5	67.74 (16)	C18—P2—C22—C24	62.07 (13)
C10—P3—C6—C5	−43.32 (16)	C19—P2—C22—C24	172.77 (11)
Ni1—P3—C6—C5	−169.94 (13)	Ni1—P2—C22—C24	−57.32 (11)
C7—P3—C6—C1	−117.58 (12)	C18—P2—C22—C23	−64.12 (14)
C10—P3—C6—C1	131.36 (12)	C19—P2—C22—C23	46.58 (14)
Ni1—P3—C6—C1	4.74 (13)	Ni1—P2—C22—C23	176.49 (10)
